# Impact of a health literacy intervention combining general practitioner training and a consumer facing intervention to improve colorectal cancer screening in underserved areas: protocol for a multicentric cluster randomized controlled trial

**DOI:** 10.1186/s12889-021-11565-3

**Published:** 2021-09-16

**Authors:** Marie-Anne Durand, Aurore Lamouroux, Niamh M. Redmond, Michel Rotily, Aurélie Bourmaud, Anne-Marie Schott, Isabelle Auger-Aubin, Adèle Frachon, Catherine Exbrayat, Christian Balamou, Laëtitia Gimenez, Pascale Grosclaude, Nora Moumjid, Julie Haesebaert, Helene Delattre Massy, Julia Bardes, Rajae Touzani, Laury Beaubrun en Famille Diant, Clémence Casanova, Jean François Seitz, Julien Mancini, Cyrille Delpierre

**Affiliations:** 1 CERPOP, INSERM UMR1295, Université Toulouse III Paul Sabatier, Inserm, UPS, Toulouse, France; 2 The Dartmouth Institute for Health Policy & Clinical Practice, Dartmouth College, Lebanon, NH USA; 3 Unisanté, Centre Universitaire de Médecine Générale et Santé Publique, Rue du Bugnon 44, CH-1011 Lausanne, Switzerland; 4 Assistance Publique - Hôpitaux de Marseille, Marseille, France; 5 Comité Départemental d’Éducation pour la Santé de Vaucluse (CoDES 84), Avignon, France; 6 EA 3279: Aix-Marseille Université, CEReSS-Health Service Research and Quality of Life Center, Marseille, France; 7 INSERM, UMR-S 1123 ECEVE Université de Paris, Paris, France; 8 INSERM, UMR 1290 RESHAPE Université Lyon 1, Lyon, France; 9 Département de Médecine Générale, Université de Paris, Paris, France; 10 Centre Régional de Coordination du Dépistage des Cancers (CRCDC-AuRA), Auvergne-Rhônes-Alpes, Saint Étienne, Cedex 02 France; 11 Faculté de Médecine - Département Universitaire de Médecine Générale, Toulouse, France; 12 Institut Claudius Regaud, IUCT-O, Registre des cancers du Tarn, Toulouse, F-31059 France; 13 P2S EA4129, Centre Léon Bérard, Université Lyon 1, Lyon, France; 14 Centre Régional de Coordination du Dépistage des Cancers d’Ile de France (CRCDC-IDF), Paris, France; 15 Institut Paoli Calmettes, SESSTIM UMR1252, Marseille, France; 16 Aix-Marseille Université, APHM, INSERM, IRD, SESSTIM, “Cancer, Biomedicine & Society” group, Hôpital Timone, Marseille, France; 17 Laboratoire CERPPS, EA 7411, Université Toulouse II Jean Jaurès, Toulouse, France; 18 Service d’Hépato-Gastroentérologie, Hôpital Timone, Assistance Publique Hôpitaux Marseille & Aix-Marseille-Université, Marseille, France; 19 Centre Régional de Coordination du Dépistage des Cancers Provence-Alpes-Côte d’Azur (CRCDC-PACA), Marseille, France

**Keywords:** Colorectal cancer screening, Health literacy, Intervention, General practitioner training, health disparities

## Abstract

**Background:**

Colorectal cancer (CRC) is a leading cause of cancer burden worldwide. In France, it is the second most common cause of cancer death after lung cancer. Systematic uptake of CRC screening can improve survival rates. However, people with limited health literacy (HL) and lower socioeconomic position rarely participate. Our aim is to assess the impact of an intervention combining HL and CRC screening training for general practitioners (GPs) with a pictorial brochure and video targeting eligible patients, to increase CRC screening and other secondary outcomes, after 1 year, in several underserved geographic areas in France.

**Methods:**

We will use a two-arm multicentric randomized controlled cluster trial with 32 GPs primarily serving underserved populations across four regions in France with 1024 patients recruited. GPs practicing in underserved areas (identified using the European Deprivation Index) will be block-randomized to: 1) a combined intervention (HL and CRC training + brochure and video for eligible patients), or 2) usual care. Patients will be included if they are between 50 and 74 years old, eligible for CRC screening, and present to recruited GPs. The primary outcome is CRC screening uptake after 1 year. Secondary outcomes include increasing knowledge and patient activation. After trial recruitment, we will conduct semi-structured interviews with up to 24 GPs (up to 8 in each region) and up to 48 patients (6 to 12 per region) based on data saturation. We will explore strategies that promote the intervention’s sustained use and rapid implementation using Normalization Process Theory. We will follow a community-based participatory research approach throughout the trial. For the analyses, we will adopt a regression framework for all quantitative data. We will also use exploratory mediation analyses. We will analyze all qualitative data using a framework analysis guided by Normalization Process Theory.

**Discussion:**

Limited HL and its impact on the general population is a growing public health and policy challenge worldwide. It has received limited attention in France. A combined HL intervention could reduce disparities in CRC screening, increase screening rates among the most vulnerable populations, and increase knowledge and activation (beneficial in the context of repeated screening).

**Trial registration:**

Registry: ClinicalTrials.gov.

Trial registration number: 2020-A01687-32.

Date of registration: 17th November 2020.

**Supplementary Information:**

The online version contains supplementary material available at 10.1186/s12889-021-11565-3.

## Background

Colorectal cancer (CRC) is a leading cause of cancer burden worldwide and the third most commonly diagnosed cancer in France. Its incidence is expected to increase by 60% to more than 2.2 million new cases and 1.1 million deaths by 2030. In France, it is the second most common cause of cancer death after lung cancer [1–3]. A mass CRC screening program was introduced in 2009. Research evidence confirms that completing a Fecal Immunochemical Test (FIT) and colonoscopy (when the FIT test result is positive) will reduce CRC related mortality [4, 5]. However, for the screening program to be effective, a 45% uptake rate is required [6, 7].

In France, the CRC screening program uptake continues to remain low (30.5% in 2018–2019), slow and socially graded [8–10]. It is significantly lower than European targets (at least 45%) [6, 7]. The national screening program, managed by regional cancer screening coordination centers (Centres Régionaux de Coordination des Dépistage des Cancers: CRCDCs) targets people between 50 and 74 years old. Standardized invitation letters are sent to eligible patients asking them to consult their general practitioner (GP) to obtain the testing kit and take the FIT test at home every 2 years. The testing kits can be given by a GP or a specialist (a gastroenterologist, gynecologist or a doctor working in French health insurance medical centers). Although the current uptake rate is only 30.5%, the objective of the French screening program is to achieve an uptake rate of between 45 and 65%. In France (as is the case worldwide), screening test uptake appears correlated with lower socioeconomic position, gender and age, with important geographic variations [8]. Screening uptake decreases with increasing levels of deprivation and is thus lower among underserved populations [11]. It is worth noting that in France and throughout Europe, high socioeconomic position, and specifically higher educational attainment, has a significant impact on reducing cancer mortality [12–14].

In this context, an important determinant of the socially graded uptake of CRC screening is health literacy (HL) [10]. HL is defined as “the degree to which individuals have the capacity to obtain, process and understand basic health information and services needed to make appropriate health decisions” [15]. HL includes: 1) basic skills in reading and writing, enabling individuals to understand health information and how to use the health system (functional HL); 2) the development of the patient’s skills in asking questions, communicating about one’s health and identifying knowledge gaps (communicative/interactive HL); and 3) the ability to make informed health decisions to appropriately manage one’s health and illness (critical HL) [16, 17]. Limited HL is associated with poorer health outcomes, poorer use of preventive health services (including cancer screening) higher burden of disease, poorer general health status, health resources use and higher mortality [18–27]. It disproportionately affects patients with financial deprivation, lower socioeconomic position, lower educational attainment and older age, thus suggesting a social gradient of HL [19, 22].

Growing research, primarily conducted in the US and UK, demonstrates the association between HL and CRC screening [27–31]. Limited HL is increasingly described as a barrier to CRC screening that significantly influences screening knowledge, beliefs and behavior [2]. People with lower HL are less likely to seek and understand information about CRC screening, and have lower self-efficacy for screening [31]. To improve outcomes and minimize health disparities, it is critical that people of lower HL and lower socioeconomic position are able to process health information, access and navigate the healthcare system to effectively manage their health and care [19, 22]. HL should be addressed and facilitated to improve equitable access to healthcare [32]. In the context of cancer prevention, addressing HL to help patients understand cancer screening can increase knowledge and screening uptake, limit social inequalities in screening and potential inequalities in survival [22, 32].

Disparities in CRC screening are widening. Physician communication to patients with limited HL skills (likely to be more socioeconomically disadvantaged) about CRC screening is often poorly understood and studied [30, 33]. GPs are frequently unaware of literacy/HL barriers that their patients may be facing. They often struggle to convey evidence-based information about CRC screening to those populations [33]. Our aim is thus to assess the impact of a HL intervention combining HL and CRC screening training for GPs with a brochure and video targeting eligible patients to increase CRC screening and improve other secondary outcomes (knowledge, patient activation, screening intention, decisional conflict, proportion of participants who complete a colonoscopy after a positive FIT test) in underserved areas of four different regions in France.

## Methods

**Fig. 1:**
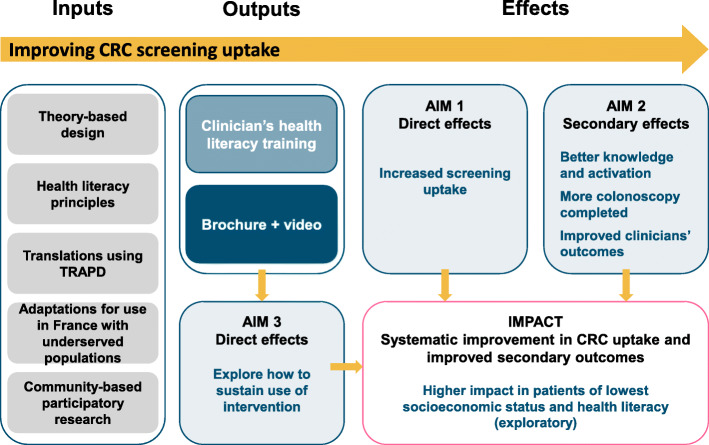
Logic model of the intervention and study. Legend: TRAPD: Translation, Review, Adjudication, Pretest, Documentation

The trial protocol follows the SPIRIT guidelines (see Additional file 1) and CONSORT statement for cluster randomized controlled trials [34, 35]. The three aims of our study are summarized in the context of the logic model shown in Fig. 1.

### Aim 1

To improve CRC screening uptake by addressing HL among underserved populations.

### Hypothesis 1.1

An interactive e-learning HL training for GPs targeting CRC screening combined with a patient-directed intervention (pictorial brochure and video) will increase CRC screening uptake (primary outcome), screening intention, knowledge, patient activation, decisional conflict and the proportion of patients who complete colonoscopy after a positive FIT test result within 1 year, among underserved populations eligible for screening.

### Hypothesis 1.2

**Fig. 2:**
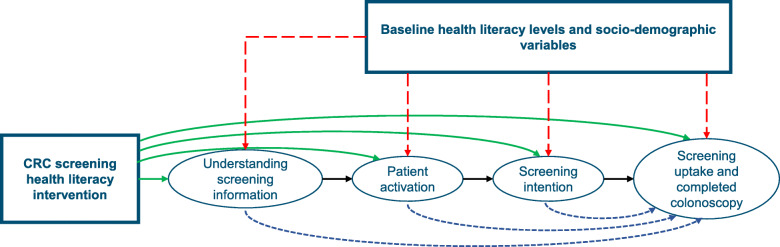
Causal model for patients enrolled in the trial. Legend: Arrows depicted in green (solid line), red (long-dashed line) and blue (short-dashed line) represent causal relationships of one variable on another. The presence of green arrows will be examined in hypothesis 1. The presence of blue arrows (mediation effects) and red arrows (moderation effects) will be examined in an exploratory analysis

The effect of the intervention on screening uptake and proportion of colonoscopy procedures completed will be mediated by post-intervention knowledge, patient activation and screening intention post consultation (see Fig. 2) after 1 year. HL level at baseline (T1) and socioeconomic position will affect screening uptake and moderate the intervention’s effect.

### Aim 2

To improve self-reported HL knowledge, skills, and behavior of the participating GPs.

### Hypothesis 2

A HL training program for GPs targeting CRC screening will improve GPs’ self-reported HL knowledge, skills, and behavior compared to baseline (collected prior to training).

### Aim 3

To understand how to promote the intervention’s sustained use and rapid implementation, using the Normalization Process Theory (NPT).

### Hypothesis 3

In accordance with NPT, a brief online HL training program will be acceptable to GPs and likely to be sustained as long as they perceive the intervention to be valuable (i.e., converting to a higher screening rate), easily integrated in their workflow (for the patient facing component of the intervention) and agree that it is important to address HL among underserved patients eligible for CRC screening.

### Design

**Fig. 3:**
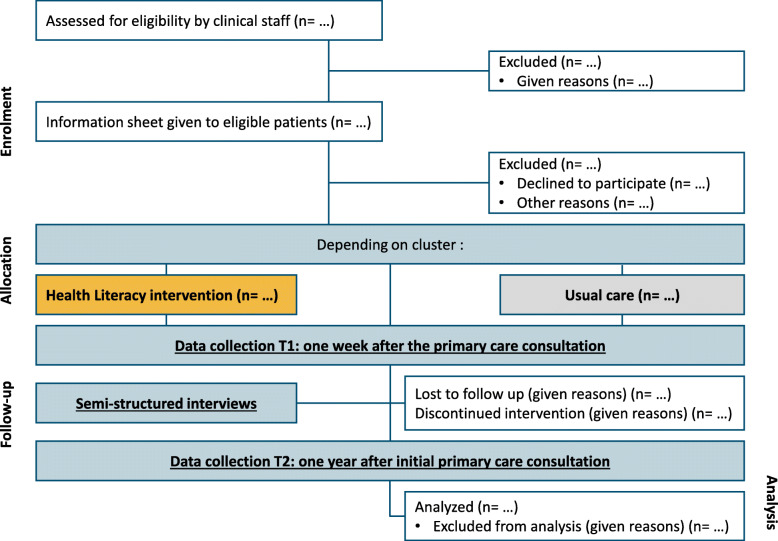
CONSORT flow diagram

We will use a multicentric two-arm (intervention versus control) randomized controlled cluster trial design (see Fig. 3). The cluster unit is the GP or the GP office (if more than 1 GP per office agrees to participate). We will target underserved areas in the following regions in France: Provence Alpes Côte d’Azur, Auvergne-Rhône-Alpes, Occitanie and Île-de-France using the European Deprivation Index (EDI) to recruit GPs working in areas with high deprivation (EDI of 4 or 5) [36]. We will follow a community-based participatory research (CBPR) approach throughout this project. We are applying CBPR principles by involving patients in all aspects of the trial, and developing effective methods for facilitating routine patient engagement (for example, with a community advisory board) [37–39]. CBPR requires partnership and shared responsibility among patients, clinicians, administrators, and nurses, and has a higher likelihood of success and implementation [38]. We have established partnerships with 10 members of the public eligible for screening in underserved areas, other professional stakeholders and organizations that support those patients (La Ligue Nationale Contre le Cancer, Santé Publique France, La Fondation ARCAD (Aide et Recherche en Cancérologie Digestive)) and the coordinators of the CRCDCs of the four regions targeted by the study. A health economic assessment will assess the cost effectiveness of the intervention.

### Conceptual model

We will use the framework developed by Cooper et al. to design and evaluate interventions designed to eliminate healthcare disparities [40]. This framework will guide the adaptation of the intervention, and will inform the design and conduct of the trial. Strategies that are likely to improve outcomes in underserved populations and reduce disparities need to be multi-factorial and address: personal factors (e.g., language, literacy, HL, education/income), as well as clinician-level factors or mediators (e.g., communication style, HL awareness, understanding of the barriers and facilitators to communicating clearly with patients of lower HL and activating/empowering them), and system-level factors. The intervention we propose to evaluate will address all three levels as follows:

- Provide accessible, evidence-based and balanced information (following plain language and HL principles) to people of lower socioeconomic position and their families/caregivers about CRC screening to improve screening uptake and other outcomes (knowledge, patient activation) (personal factors);
- Address HL by training GPs in HL principles and CRC screening (clinician-level factors or mediators);
- Standardize the information provided to all patients and improve patient activation to improve screening uptake, overall health and disease management, and the proportion of patients who complete a colonoscopy after a positive FIT test (system-level factors) in the four selected regions.

In parallel, we will also be guided by the Medical Research Council (MRC) framework for the evaluation of complex healthcare interventions [41]. We have successfully used those frameworks before [42].

### Participants

#### Inclusion criteria

Participants will be invited to participate and included if:

- they are aged between 50 and 74 years old (age group eligible for CRC screening);
- they are eligible for CRC screening, have health insurance, and are seen by a participating GP in one of the four included regions;
- they are able to complete a questionnaire in French either alone or with help from a caregiver or relative, or in another language assisted by an interpreter.

Individuals with an intellectual disability will be included as long as they are able to complete a questionnaire alone or with help from a caregiver or relative.

#### Exclusion criteria

We will exclude patients not eligible for CRC screening and those whose mental health status precludes participation in the study as determined by the participating GP or a qualified staff member.

### Setting

The study will be conducted in underserved geographical areas in and around the cities of Toulouse, Lyon, Paris and Marseille. We will use the European Deprivation Index (EDI) to identify GPs in disadvantaged areas (EDI 4 or 5) [36].

### GP recruitment

To recruit GPs, we will first use a purposive sampling approach to include a balance of male and female GPs, practicing alone and in medical centers, in each region. GP work addresses will be coded against the EDI to establish if their practice covers a geographically disadvantaged area (EDI 4 or 5). GPs will be approached by email or telephone and given information about the trial. If this approach fails, we will transition to a convenience sampling approach, using contacts in our research network. Confirmation of participation will be by email and further contact details obtained. GPs will be randomly allocated to the control or intervention arm prior to the intervention training process, 2 months before patient recruitment begins. If a participating GP leaves their practice or decides to leave the trial, we will recruit another GP and assign him or her to the same arm.

### Participation and recruitment procedures for trial entry

Within the 4 regional areas, clinical research assistants (CRAs) will work in collaboration with recruited GPs. Participants will be approached in primary care medical centers or at individual GPs’ offices by their doctor, a resident (“interne”), or another member of the medical center. Clinical staff will confirm eligibility (see Fig. 3) and obtain verbal consent. Once participants are recruited, CRAs will re-confirm verbal consent over the telephone and check whether they are able to complete the questionnaires alone or if they need assistance.

### Ethical approval, consent and recruitment strategies

The trial has received approval from the local research ethics committee, the Comité d’Ethique de la Recherche (CER) at the University of Toulouse III Paul Sabatier, France (ref 2021–349, dated 8th March 2021). Eligible participants will be asked to read a brief information sheet written in plain language or have a resident, mediator/patient navigator or other member of medical staff read the information out loud to them. This is particularly relevant since participants targeted by the trial are likely to have lower HL and lower textual literacy. According to the French health research classification, the ethics committee’s requirement for consent for this study requires participants’ verbal consent only or ‘non-opposition’ consent. Participants recruited by an intervention GP will be given a pictorial brochure about CRC screening and asked to watch a 3-min video on a tablet before exiting the primary care center/GP’s office. Participants recruited by a GP in the control arm will discuss the trial and CRC screening in their usual way.

One week after (T1), the CRA will call participants and offer to conduct a standardized telephone interview where the questions will be read out loud to them. Whenever possible, T1 data collection will be carried out in a single phone call. If the participant does not answer the first telephone call, the CRA will make three further attempts to contact them and leave up to three voice messages. CRAs will verify telephone contact details with the recruiting GP practices. Given participants are likely to have lower HL, this is the best strategy to maximize retention and minimize participant burden and missing data.

One year after (T2), CRAs will call participants for the follow-up assessment and offer to conduct a further standardized telephone interview. As with T1, attempts to verify contact details and to call the participant several times will be made.

### Intervention

We will translate and adapt (using a translation procedure adapted from TRAPD) an intervention previously developed and evaluated in a randomized controlled trial conducted in the US to address HL and improve CRC screening [33]. This intervention combined a 2-h HL and CRC screening training targeting GPs and a patient level intervention consisting of a short brochure and video. To maximize generalizability and implementation, we will develop a 2 h e-learning HL training in French and a one-hour booster session similar to the previous trial’s intervention [33]. The first hour will be an online course that will primarily include didactic teaching using videos. The second hour will consist of a c-MOOC (Connectivist – Massive Online Learning Course), an online classroom model, where all participating GPs in the intervention group can interact with each other and with a trained facilitator from our team, experienced in HL training (M-AD). This interactive session will build on the content provided in the initial didactic session. The c-MOOC is based on a learner-driven dialogue where participation, discussions and reflection on the content presented are the intended focus. It will also include small group discussions and role-playing sessions focused on empowering GPs to discuss CRC screening effectively with their patients. A 1-h c-MOOC booster training session will be held 6 months later. During this session, GPs will receive data on their screening uptake rates over the previous 6 months, a reminder of practical strategies to communicate with patients with lower HL skills in busy, high-volume primary care practices. They will also have the opportunity to engage in small group discussions. Each GP in the intervention arm will receive 150 euros for completing the first 2-h learning training (1 h didactic learning + 1 h c-MOOC) and 75 euros for completing the booster session (1 h c-MOOC).

The patient-facing intervention (brochure and video) will follow key plain language and HL principles to translate evidence-based information in to content that all patients can understand. Images and simple text will be used to facilitate understanding and promote informed choice and patient-centered care for all patients, irrespective of HL levels. To promote accessibility among underserved populations whose primary language may not be French, we will use existing systems already embedded in our teams in each region and additional interpretation services as relevant.

### Outcome measures

#### Primary outcome measure

The primary outcome measure is CRC screening uptake assessed 1 year after the initial recruitment consultation (T2) by self-report questionnaire (see Table 1). Each CRCDC, in collaboration with the health insurance body (CPAM) in each region, will also provide data on CRC screening uptake at 6 months (for feedback to GPs in the intervention arm) and 1-year post enrollment.

**Table 1:** Outcome measures and data collection timepoints for patients

Outcome measures	Timepoints	
	(T1) One week after the primary care consultation where CRC screening is discussed	(T2) 1 year after the CRC screening consultation
Number of eligible patients identified and successfully enrolled	**X**	
Number of patients enrolled who receive the patient-facing intervention	**X**	
Discontinuation rate		**X**
CRC screening uptake (primary outcome) with self-report item		**X**
Screening intention (1 items)	**X**	
Knowledge of CRC screening (2 items)	**X**	**X**
Patient activation (13 items)	**X**	**X**
Health literacy (7 items)	**X**	
EPICES (11 items)	**X**	
SURE (4 items)	**X**	
Was the questionnaire completed alone? (1 item)	**X**	**X**
Demographics (9 items)	**X**	
**Total number of self-report items per timepoint**	**48**	**17**

#### Secondary outcome measures

We will use standard telephone interviews with patients to collect secondary outcomes measures 1 week (T1) and 1 year (T2) after the initial recruitment consultation. These include: screening intention (1 item, assessed at T1), knowledge about the test instructions and the objectives of the screening test, (2 items, assessed at T1 and T2), patient activation (13 items- assessed at T1 and T2). We will use a validated short form patient activation measure, already available in French [43, 44]. We will assess HL using the Newest Vital Sign [45] (6 items) and the “single item literacy screener” [46] (1 item) (both assessed at T1). We will evaluate decisional conflict with SURE (4 items, assessed at T1) [47]. Finally, we will assess the proportion of patients who complete colonoscopy after a positive FIT test result within 1 year (collected by each regional coordination center in collaboration with the health insurance body). We will also collect socio-demographic information: age, gender, comorbidities, mother tongue, marital status, length of time living in France, educational attainment, income bracket, whether help was required to complete the questionnaire, any previous screening tests completed and the EPICES (Évaluation de la Précarité et des Inégalités de santé dans les Centres d’Examens de Santé), a validated measure of deprivation and social health in French, with 11 self-reported items (assessed at T1) [46].

Secondary outcomes for GPs will include a translated 12-item questionnaire assessing self-reported HL knowledge, skills, and behavior, collected prior to the intervention training and 1 year post training for GPs in both trial arms. We will also collect demographic information: age, gender, type of practice (GP practicing alone or in a medical center), year of medical training and participation in (and details of) a lump-sum participation package, ROSP (la rémunération sur objectifs de santé publique), an incentivized public health initiative. Tables 1 and 2 summarize the outcome measures and data collection timepoints.

**Table 2:** Outcome measures and data collection timepoints for GPs

Outcome measures	Timepoints	
	Immediately before the health literacy training	1 year after the health literacy training
Health literacy knowledge, skills, and behavior (12 items)	**X**	**X**
Demographics (6 items)	**X**	
**Total number of self-report items per timepoint**	**18**	**12**

### Sample size and power calculation

We plan to recruit at least 32 GPs across 4 regions. Based on Ferreira et al.’s trial findings, to detect a 15% difference between the control and intervention arm in CRC screening uptake, with an intra-cluster correlation (ICC) of 0.004 and a 25% attrition rate, a sample size of 1000 people eligible for screening is required [33]. This 15% difference is conservative. The ICC is based on previous studies similar to this trial. Each clinician will be expected to recruit approximately 32 patients, or as many eligible patients as possible if they cannot recruit 32, over the course of 1 year. On average, French GPs have about 300 eligible patients for CRC screening each year. Targeting 32 patients of about 300 represents just under 10%, which is achievable.

### Randomization

#### Sequence generation, type of randomization and allocation concealment

We will use an R script written by the trial statistician to perform the GP randomization. The random allocation sequence will be concealed until interventions are assigned. We will use block randomization to ensure a balanced allocation of each cluster (GP or GP office) in each region to the intervention and control arms. Participants recruited by GPs will therefore be a priori allocated to either the control or intervention arm by virtue of the GP that has recruited them. GPs will not be required to assess HL or deprivation levels (as these are not a inclusion or exclusion criteria) as the GP practice itself has already been identified as being located in a disadvantaged area.

#### Minimizing differential recruitment

To minimize differential recruitment, which can often occur in cluster randomized trials [48, 49], recruitment numbers in both arm of the trial will be routinely monitored at monthly team meetings. Once the target number of participants in each arm has been reached, recruitment will be stopped.

### Changes to intervention allocation

There are no established criteria for discontinuing or modifying the allocated intervention for study participants due to the low-risk nature of the trial. However, each CRA will be asked to record the reasons for patient refusal to participate/discontinuing at each stage of the study and to take field notes.

### Blinding

Due to the nature of intervention delivery, it will not be possible to blind GPs to their allocation to either the control or intervention arm. At the individual level, patients invited to participate in the trial will likely be unaware that they are in the intervention or control arm. Furthermore, the CRAs collecting participants’ primary outcome data, assessing outcomes and conducting the analyses will not know to which group participants were allocated. The data analyst will also be blinded to arm allocation.

### Qualitative data

Following recruitment, we will conduct semi-structured qualitative interviews with a purposive sample of up to eight health professionals in each region (up to 24, based on thematic data saturation) and at least six patients per region (up to 48, based on thematic data saturation). The aim of the interviews is to explore strategies that promote the intervention’s sustained use and rapid implementation, using NPT [50] which has been successfully used before [51, 52].

### Health economic assessment

A cost-effectiveness analysis will be conducted to assess the medico-economic impact of implementing the intervention among patients with lower HL in disadvantaged areas of the four targeted regions. The purpose of this analysis is to compare the costs related to the effectiveness of the intervention with health professionals (the 2-h HL training) and users (brochure and video) versus usual care (control arm). First, we will calculate the cost per additional CRC screening performed by dividing the difference between the costs of implementing the intervention and the cost of the usual care group, relative to the observed increase in testing in the intervention group. Second, we will calculate the cost per new cancer diagnosed in patients with a positive screening test. The time horizon of the analysis will be the duration of the study (1 year).

### Data management and statistical analysis

### Data management

All data will be anonymized using ID codes for both GPs and participants. All data will be managed securely via MS Access databases, linked across the four sites via the secure data sharing system SCOUT, hosted by the University of Toulouse III Paul Sabatier. Access to data will be granted only to members of the study team. Data from questionnaires and interview transcripts will be kept for a period of 2 years after publication of the results is completed.

All data will be verified on entry into the systems and cleaned. An audit process of typically 10% of the data will also be completed on the T1 data prior to the T2 data being collected.

### Statistical analysis

Initial examination of data will include descriptive statistics, frequency distributions, and histograms to identify outliers and missing data. The baseline data in each arm will be compared to ensure randomization was conducted successfully. We will use the Stata 16 software (Stata corp, US) to perform all analyses.

All participants will be asked to indicate at T1 if they have received the intervention to enable an intention-to-treat (ITT) and as-treated analysis. We will adopt a regression framework for all analyses as it allows seamless transition between basic analyses involving a single predictor and more complex analyses involving additional predictors (mediation variables, control covariates, time-trends, interaction terms or effect modifiers).

Further, the regression framework allows clustering of observations due to repeated measurements on patients across time, nesting of health professionals within sites, and patients within health professionals, to be accurately accounted for using mixed-effect regression models [53] or generalized estimating equations [54, 55].

### Analyses corresponding to aim 1

We will first perform separate analyses for each data collection period (T1 and T2) using linear and logistic regression models as appropriate for continuous (knowledge, patient activation, HL), and binary (screening intention, screening uptake, colonoscopy completed) outcomes respectively. The results will provide potentially valuable insights into how rapidly the intervention affects outcomes. Outcomes measured twice may also be analyzed using a longitudinal model. A secondary analysis that adds predictors for the number of patients seen by the healthcare professional in the intervention arms will examine whether there are physician learning effects. To gain insight into whether the intervention will be more effective according to the patient’s HL level and socioeconomic position, we will test the first order interaction between HL level/socioeconomic position and the intervention indicator variables. EPICES scores will also be used.

### Analyses corresponding to aim 2

We will use exploratory mediation analyses. The aim is to identify and explicate the mechanisms or processes that underlie the relationship between the intervention and a dependent variable via the inclusion of a third explanatory variable, known as a mediator variable (e.g., knowledge, patient activation). We are specifically interested in whether interventions operate through the mediator as opposed to directly affecting the outcome. To determine the generalizability of these mechanisms and identify subpopulations for whom mediation is most pronounced, we will compare the mediation effects across different subgroups (e.g., higher HL versus lower HL).

### Analyses corresponding aim 3

We will use a framework analysis, guided by Normalization Process Theory (NPT) [50], having successfully used this approach previously [51, 52]. NPT was developed to understand how complex interventions become implemented in routine healthcare settings. NPT was built around four theoretical constructs: 1) Sense-making or coherence: processes of individual and communal sense-making of a complex intervention regarding its use and value 2) Participation: processes of ‘cognitive participation’ that promote or hinder users’ buy-in and commitment to the intervention 3) Action: processes of ‘collective action’ that determine or hinder whether the intervention is being used by all as intended 4) Monitoring: processes of communal and individual appraisal of the effect of the intervention. We will use NPT as an analytical lens to consider the data collected according to our hypothesis. Observations and field-notes taken by the CRAs during the recruitment process will also be included in the analysis. Initial descriptive codes will be generated based on the four NPT constructs. In-vivo coding will also be used to capture other naturally occurring exchanges. Categorical codes that group initial and in-vivo codes will be developed in a third round of coding.

### Missing data

Most data collection will be via standardized telephone interview (at T1 and T2), which provides opportunities for preventing and monitoring missing data. We will investigate whether multiple imputation is required to cope with any missing baseline, interim, and outcome data.

### Heterogeneity of treatment effect (HTE)

As the HTE analyses are mostly exploratory rather than hypothesis-driven, exploratory subgroup analyses will be conducted to identify hypotheses for future evaluation. Patient characteristics will be considered for treatment by covariate interactions and include socioeconomic position, educational attainment, age, gender and HL.

### Community advisory board

Community advisory board members were involved in the design and planning of the study. Their tasks consisted of participating in the development of the pictorial brochure and to verify the accessibility of the language used for target patients. When the time comes, they will also contribute to the dissemination of the study and its results through their networks. They were chosen because of their experience with CRC, as a patient advocate or working in an organization promoting cancer prevention. They are invited to meetings every 3 months by video conference and compensated for their attendance with gift cards. This meeting occurs in parallel to the Trial Steering Group meetings.

## Discussion

Our patient and professional stakeholder partners, as well as all four CRCDCs have emphasized the critical importance of addressing health inequalities in CRC screening uptake and the originality of this approach. This study is the first to address HL training for French GPs in a country with a low screening rate and growing health inequalities. The question of inequity is central to this protocol and to the delivery of the intervention. Health inequalities are a strong determinant of lower screening uptake and increased CRC mortality. Following a proportionate universalism approach, the intervention we propose to evaluate targets HL among underserved population to address health inequalities and improve CRC screening uptake and other outcomes [56].

Limited HL and its impact in the general population and in underserved groups is a growing public health and policy challenge in Europe. Addressing HL and intervening to mitigate it and improve the prevention of cancers is in its infancy in France. If addressed, it could reduce disparities in CRC screening, increase screening rates among the most vulnerable populations, and increase knowledge and activation.

To disseminate the findings, we will work with multiple partners including La Ligue Nationale Contre le Cancer, Santé Publique France and the ‘College National des Généralistes Enseignants’ to distribute and implement the intervention. We will also work with the CRCDCs and their public health and GP coordinators to understand how the training could become part of continuing professional development for practicing GPs. To promote generalizability, large-scale diffusion, dissemination and sustained use beyond the funded-research period, we have purposefully decided to use a blended e-learning HL training. This intervention could thus be disseminated easily, on a national scale.

We anticipate that the study outcomes have the potential to change the way GPs inform and support all patients about CRC screening, and particularly those who are underserved with lower HL. The study findings will be beneficial to clinicians, policy makers and other national and community stakeholders who aim to improve CRC screening across socioeconomic strata to reduce health inequalities. The findings will directly benefit patients, their families and caregivers, as well as inform academics and others who strive to produce interventions that are beneficial to all and can be effectively implemented in routine care.

## Supplementary Information

**Additional file 1.** Completed SPIRIT guidelines checklist for this DECODE trial protocol article.

## Data Availability

Data sharing is not applicable to this article as no datasets have yet been generated or analyzed.

## Abbreviations

CER: Comité d’Ethique de la Recherche (research ethics committee)
c-MOOC: Connectivist - Massive Online Learning Course
CPAM: Caisse Primaire d’Assurance Maladie
CBPR: Community-Based Participatory Research
CRC: Colorectal cancer
CRCDC: Centre Régional de Coordination du Dépistage des Cancers (Regional cancer screening coordination center)
CRA: Clinical research assistant
EDI: European Deprivation Index
FIT: Fecal Immunochemical Test
GP: General practitioner
HL: Health literacy
HTE: Heterogeneity of treatment effects
ICC: Intra-cluster correlation
ICMJE: International Committee of Medical Journal Editors
MOOC: Massive Open Online Course
NPT: Normalization Process Theory
